# Knowledge, Attitude, and Practice of Medication Use During Pregnancy: A Cross‐Sectional Study in Western Uganda

**DOI:** 10.1002/hsr2.70644

**Published:** 2025-04-10

**Authors:** Musiime Brian, Narayana Goruntla, Bhavana Reddy Bommireddy, Bhavani Mopuri, Easwaran Vigneshwaran, Mohammad Jaffar Sadiq Mantargi, Vishnuvandana Bandaru, Joseph Obiezu Chukwujekwu Ezeonwumelu, Sarad Pawar Naik Bukke, Tadele Mekuriya Yadesa, Ebere Emilia Ayogu

**Affiliations:** ^1^ Department of Clinical Pharmacy and Pharmacy Practice, School of Pharmacy Kampala International University, Western Campus Ishaka Uganda; ^2^ Department of Pharmacy Practice Raghavendra Institute of Pharmaceutical Education and Research (RIPER)—Autonomous Anantapur Andhra Pradesh India; ^3^ Department of Clinical Pharmacy, College of Pharmacy King Khalid University Abha Kingdom of Saudi Arabia; ^4^ Department of Pharmaceutical Sciences, Pharmacy Program Batterjee Medical College Jeddah Kingdom of Saudi Arabia; ^5^ Department of Pharmaceutical Analysis Balaji College of Pharmacy Anantapur Andhra Pradesh India; ^6^ Department of Pharmaceutics and Pharmaceutical Technology, School of Pharmacy Kampala International University, Western Campus Ishaka Uganda; ^7^ Department of Clinical Pharmacy and Pharmacy Management, Faculty of Pharmaceutical Sciences University of Nigeria Nsukka Nigeria

**Keywords:** attitude, knowledge, medication safety, practice, pregnant women, Uganda

## Abstract

**Background:**

Medication use (MU) during pregnancy can increase the risk of maternal and fetal health consequences. Women's knowledge, attitude, and practice (KAP) regarding safe MU can influence pregnancy outcomes. The study aimed to assess and explore women's KAP regarding safe MU during pregnancy and identify possible determinants.

**Methods:**

A cross‐sectional analytical, interview‐based survey was conducted among pregnant women attending antenatal care (ANC) visits in the obstetrics and gynaecology department of Kyegegwa General Hospital, Kyegegwa district, Western Uganda. A 24‐item pre‐designed, validated structured questionnaire was used to assess the KAP of MU among pregnant women. Binary and multiple logistic regression analyses were used to identify factors associated with KAP about MU.

**Results:**

A total of 415 pregnant women with a mean (SD) age of 26.51 (5.15) years were included. Most of the women have a positive attitude (78.60%) towards safe MU, but less than half only hold adequate knowledge (42.60%), and rational practice (34.50%). Factors such as secondary education level or less (AOR = 0.15; 95% CI = 0.01–0.38), urban residence (AOR = 1.54; 95% CI = 2.68–4.49), profession (AOR = 1.94; 95% CI = 2.36–10.59), regular ANC visits (AOR = 1.22; 95% CI = 1.06–3.48), GP visit during pregnancy (AOR = 2.54; 95% CI = 1.09–5.91), and using at least one medication (AOR = 9.00; 95% CI = 2.78–6.43) were significantly associated with adequate knowledge regarding MU. The practice domain revealed that age less than 30 years (AOR = 0.53; 95% CI = 0.28–0.98), profession (AOR = 2.56; 95% CI = 1.86–7.59), regular ANC visits (AOR = 1.79; 95% CI = 1.05–3.74), and GP visit during pregnancy (AOR = 1.73; 95% CI = 1.02–3.25) were significantly associated with rational practice.

**Conclusion:**

The study concludes that three‐fourths of the pregnant women have a positive attitude regarding safe MU, still there is an extensive gap in transforming this positive attitude into rational practice due to lack of adequate knowledge. So, to address the gap identified in the knowledge and practice domain, hospital‐based educational interventions on safe MU can be initiated by targeting young age, lower or no education, rural residents, and participants working in non‐healthcare profession.

## 1 Introduction

Pregnancy is a dynamic process in which the woman's physiology modifies to meet the metabolic needs of the growing fetus [1]. These changes can affect the drug's pharmacokinetics and placenta transfer, which causes harm to the fetus [2]. When using medicine during pregnancy, it is crucial to thoroughly evaluate the potential benefits and risks to pregnant women and their unborn babies [3].

Evidence shows that the use of prescription or non‐prescription medicine among pregnant women has increased dramatically in recent years around the world [4, 5]. Medication use (MU) during pregnancy cannot be completely avoided due to underlying diseases like diabetes, hypertension, asthma, and epilepsy, which require ongoing or episodic treatment. Furthermore, medicine is recommended to treat pregnancy‐induced medical conditions (anemia, pre‐eclampsia, eclampsia, gestational diabetes, hypothyroidism, migraine, headache, hyperacidity, nausea, and vomiting) that require pharmacological intervention [2]. Although the use of medications during pregnancy is common and unavoidable, information about their safety is limited and unreliable due to a lack of clinical trial evidence [6]. Thus, inappropriate medication use during pregnancy can have negative consequences for both the mother and the fetus [7]. In this context, rational medication use during pregnancy is critical for public health and should be prioritized by health policymakers.

It has been documented that teratogenic drugs cause 1% of all congenital abnormalities [8]. Though it is less, uncertainties about fetal well‐being may lead to medication non‐adherence in pregnant women, resulting in additional harm to the fetus and women due to uncontrolled pre‐existing disease conditions. On the other hand, a lack of knowledge about safe medication use during pregnancy would result in self‐medication, inappropriate medication use, and adverse outcomes such as fetal abnormalities, abortion, maternal bleeding, and even death [9]. The Food and Drug Administration (FDA) classified drugs into five categories to promote safe medication use in pregnancy: A, B, C, D, and X. In this classification, Category A is considered the safest medicine, while Category X is contraindicated during pregnancy [10].

Studies have evaluated the knowledge, attitude, and practice of pregnant women regarding medication use in pregnancy across the world, including in Nigeria, Eritrea, Italy, Tanzania, Malaysia, USA, Serbia, Norway, Saudi Arabia, and India [2, 3, 11, 12, 13, 14, 15, 16, 17, 18]. All of these studies revealed varying levels of knowledge, attitude, beliefs, and practice regarding medication use during pregnancy.

In developing countries, poor pregnant women's health‐seeking behavior, delayed antenatal care (ANC) initiation, low maternal educational status, lack of access to updated medicines information, poor access to health facilities, and a lack of training for health‐care providers may all contribute to the issue of drug safety in pregnancy [19]. According to a recent study conducted in Uganda, 44% of pregnant women took at least one potentially harmful medicine [20]. Antimicrobials are the most prescribed medications, while analgesics are the most frequently used over‐the‐counter medications in pregnant women. The study found that presence of a co‐morbidity and taking more medications have high risk toward consumption of a harmful medicine during pregnancy. The above evidence suggests that there is a need to reduce the consumption of harmful medicine during pregnancy. This can be achieved by providing educational interventions to improve the KAP of safe medication use during pregnancy. A study conducted in Gulu district of Uganda shows that the prevalence of herbal medicine use among pregnant women was 20%, and it recommends to improve the knowledge regarding safe herbal medicine use during pregnancy in Uganda [21]. To the best of our knowledge, other researchers did not pay attention to address the level of knowledge, attitude, and practice regarding medication use during pregnancy in Uganda. The current study focuses on to estimation of the level of KAP regarding safe medication use and associated factors during pregnancy. Every pregnant woman must have optimal knowledge and a positive attitude toward medication use to avoid OTC or self‐medication practice [22]. Medication use under the supervision of a medical practitioner will reduce the occurrence of drug‐induced birth defects and pregnancy‐related complications [23]. The information generated from this could be useful for developing strategies to fill the gaps and to support pregnant women in making informed decisions about safe medication use. Therefore, the study aimed to assess and explore women's KAP regarding safe MU during pregnancy and identify possible determinants.

## 2 Methods

### 2.1 Study Design and Setting

An interview‐based, cross‐sectional survey was conducted among pregnant women attending antenatal care (ANC) in the obstetrics and gynecology department of Kyegegwa General Hospital, Kyegegwa district, Western Uganda. An average of 100 attendants in ANC per week and 140 attendants in the maternity ward per month was documented in this facility. Apart from dealing with obstetrics and gynecological disorders, the hospital also offers pediatric, surgical, medical, psychiatric, intensive care, dental, accident and emergency, ENT, HIV care, and other ancillary healthcare services. The study was conducted for 6 months, spanning from October 2023 to March 2024. The study was conducted following the STROBE (Strengthening the Reporting of Observational Studies in Epidemiology) guidelines.

### 2.2 Study Participants

All pregnant women who visited the antenatal care unit for routine checkups during the study period were included after providing oral and written informed consent. Pregnant women with labor pain, critical illness, unconsciousness, or inability to respond to oral instructions were excluded from the study.

### 2.3 Ethical Considerations

The study protocol, survey instrument, and informed consent process were approved by the Kampala International University—School of Pharmacy Research Committee (KIU SPRC 003/24). The researcher explained the study protocol and objectives to the eligible participants and received oral and written consent voluntarily. All participants had the right to withdraw from the study at any point during the research process (consent, initiation, process, and completion). The study followed the guidelines of the Declaration of Helsinki 1975 for research on human volunteers.

### 2.4 Sample Size Estimation and Sampling Method

The sample size was estimated using a single population proportional formula $\left(n=Z^{2}P(1‐P)/d^{2}\right)$, with the assumption that 50% (*P* = Prevalence) women were aware of the safe use of medication during pregnancy [24]. By setting a 5% (95% CI: 45%–55%) margin of error (*d*), 80% power of the study, and 1% design effect, the sample size was determined as 384. By assuming 5% of the nonresponse rate the final sample size was estimated as 404. Convenience sampling, a non‐probable sampling strategy, was employed to select study participants who fulfilled the eligibility requirements. Though the convenience sampling can result in sampling bias which can affect the generalizability of the findings, we adopted this technique to have maximal sample size within the duration of academic research, and easiness in getting samples from resource limited settings.

### 2.5 Development and Validation of Survey Tool

An extensive literature search was performed in PubMed/Medline to identify the previous studies relevant to the assessment of KAP towards medication use during pregnancy. A total of 8 studies were used from the literature to frame the questions and modified according to the hospital drug policy on rational medicine use in pregnancy [25, 26, 27, 28, 29, 30, 31, 32]. Pregnant women's medical illnesses and obstetric characteristics can significantly influence their medication use. As per the hospital's drug policy, the survey tool included obstetric, clinical, and medication use variables that could change the KAP of medication use during pregnancy. The prepared questionnaire was subjected to content validation by the expert panel composed of one obstetrician, one clinical pharmacist, one physician, one epidemiologist, and one pediatrician. The questionnaire has 8 knowledge, 10 attitude, and 6 practice questions. The content validity index (CVI) was calculated for knowledge, attitude, and practice questions by assigning expert opinions on a 4‐point Likert scale. Knowledge, attitude, and practice CVIs were 0.95, 0.83, and 0.89, respectively (≥ 0.80 considered acceptable). A pilot study was conducted among 30 eligible pregnant women to determine the questionnaire's internal consistency. The reliability test results from a pilot sample survey revealed Cronbach's alpha values of 0.88, 0.91, and 0.89 for knowledge, attitude, and practice domains that are reliable to use.

### 2.6 Survey Tool

The survey tool consists of four sections: (1) demographics, obstetrics, and medical profile; (2) knowledge towards medication use during pregnancy; (3) attitude towards medication use during pregnancy; and (4) practice towards medication use during pregnancy.

### 2.7 Demographics, Obstetrics, and Medical Profile

Demographic variables like age, nationality, educational status, occupation, and residence were included in the survey tool. The survey tool also comprises obstetrics characteristics like gestational age, gravida, number of antenatal visits, and current pregnancy risk category. Medical characteristics like self‐reported health problem and medical consultation in the last year, drug use in previous pregnancy, current medical illnesses and consultations during pregnancy, current drug therapy, medication use type (OTC or prescribed), were included in the data collection form.

The number of antenatal visits were categorized in to regular if women adhere to the WHO recommendation that women should have first contact in the first 12 weeks of gestation, with subsequent contacts takes place at 20, 26, 30, 34‐, 36‐, 38‐ and 40‐weeks' gestation [33]. If any women not met above criteria would be categorized as irregular to ANC visits. We collected the pregnancy risk from patient medical records, where health care providers provided information about high‐ or low‐risk pregnancy.

### 2.8 Knowledge of Medication Use During Pregnancy

Eight questions related to the name of the drug, an indication of the drug, safety, medicines needed to be consumed, a critical period for drug effects on the fetus, effects of unwanted drug use, and tailoring medication regimen in chronic disorders were included to assess the knowledge toward safe medication use among pregnant women. Though questions have dichotomous response like “Yes” or “No”, participants were enquired about mentioning of the answer if they opt “Yes”. All answers obtained from the pregnant women were screened fort the correctness. In the knowledge domain, each correct response was given a score of one, and the wrong answer was zero which gives a maximum score of 8 and a minimum score of zero for each participant. Based on the final score gained in the knowledge domain all subjects were classified to have adequate (> 50%; score > 4), and inadequate (≤ 50%; score ≤ 4) knowledge.

### 2.9 Attitude Toward Medication Use During Pregnancy

The attitude domain comprises 10 statements about women's perception that all medicines are harmful to the fetus and mother, stoppage of medicines is essential for better fetal growth, high use of medicines, untreated illness may increase the risk of fetal growth, medicine intake is safe, it is essential to take advice from the pharmacist or doctor before taking medicines, and safe use of prescription medicine, OTC medicine, natural medicines, vitamins, supplements, and topical preparations during pregnancy.

In the attitude domain, each statement regarding medication use during pregnancy was scored on the 3‐point Likert scale ranges Disagree = 3, Uncertain = 2, and Agree = 1. Whereas, statements third, fourth, sixth, and eighth were reversely scored. Each participant acquires a score maximum of 30 and a minimum of 10 in the attitude domain. If the women scored more than or equal to 15 were categorized as having a positive attitude, and less than 15 was a negative attitude towards safe medication use during pregnancy.

### 2.10 Practice of Medication Use During Pregnancy

Safe medication use practice among pregnant women were assessed by using six questions related to medication intake after consulting a physician or pharmacist, reading medicine leaflets, regular doctor visits, asking about the purpose and safety of medicines while prescribing and dispensing, and using evidence‐based medicine resources. Pregnant women are expected to get opinion from the doctor or pharmacist about medicine information or intake under sources of information. In the practice domain, each correct response was given a score of one, and the wrong answer was zero which gives a maximum score of six and a minimum score of zero for each participant. Based on the score obtained in the practice domain, women were categorized into rational (= 6), and irrational (< 6) practice toward safe medication use during pregnancy.

### 2.11 Data Collection

The research goals, protocol, and expected outcomes were clearly explained to eligible pregnant women before obtaining consent for participation. A total of 453 eligible pregnant women were approached at the ANC outpatient unit to participate in the current study. Among 453 pregnant women, 20 refused to register, and 18 did not respond due to lack of time. During the study frame, a face‐to‐face interview was conducted among 415 pregnant women. The interview lasted an average of 15–20 min. A predesigned and validated data collection survey tool was used to assess the KAP toward medication use during pregnancy. The required data was collected from medical records and interview of the participants by using data collection survey tool. The principal investigator checked the accurateness of the data abstraction process according to the research protocol. On site, a quality check was performed every day to remove errors made in the data collection procedure. The collected data was double checked for completeness, clarity, and consistency by the principal investigator and data collector. If any missing data was observed in the data collection process, it was not subjected for further data analysis.

### 2.12 Data Analysis

IBM SPSS software for Windows, version 22.0 (IBM Corp., Armonk, NY, USA) was used to analyze data collected from the study participants. Before commencing the analysis, the data underwent cleaning, sorting, and processing within an Excel spreadsheet. Descriptive statistics like frequency, proportion, mean, and standard deviation were used to represent the socio‐demographics, obstetrics, clinical, medication use profile, knowledge, attitude, and practice toward medication use by pregnant women. The study employed binary and multiple logistic regression analyses to examine the relationships between the independent variables (socio‐demographics, obstetrics, clinical, and medication use profile) and the dependent variable (knowledge, attitude, and practice about safe medication use during pregnancy). The rationale behind the selection of proposed variables in the regression model was based on the influence of variables on medication use practice during pregnancy. All significant variables with *p*‐values less than 0.20 in the binary logistic regression analysis were included in the multiple logistic regression model [34]. A two‐way *p*‐value of less than 0.05 was considered statistically significant.

## 3 Results

We identified 415 pregnant women who met our inclusion and exclusion criteria. The mean age of pregnant women was 26.51 years (SD: 5.15 years). Most of the women are aged between 21 and 30 years (278; 66.99%), Ugandan (397; 95.66%), have a tertiary/university level of education (164; 39.52%), housewives (283; 68.17%), and residing in a rural area (258; 62.17%). The obstetric profile of the women shows that the majority are in the second trimester (203; 48.91%), gravida two (224; 53.97%), not attending a scheduled number of ANC visits (312; 75.18%), have non‐risky pregnancy (357; 86.02%), and no experience of stillbirth or miscarriage (370; 89.16%). Clinical and medication use characteristics shows that most of the pregnant women are not suffering with any medical illnesses (297; 71.56%), not visited GP in the current pregnancy (351; 84.58%), not experienced any health consequence in the last year (389; 93.73%), using medicines in the current [(One = 47; 11.32%; Two = 133; 32.01%; Three or more = 136; 32.77%)] and previous pregnancy (286; 68.91%) and not using OTC medicines (297; 71.57%). We described the demographics, obstetrics, clinical and medication use characteristics of the pregnant women in Table 1.

**Table 1:** Demographics, obstetrics, clinical, and medication use characteristics of the study participants (*n* = 415).

Variable	Frequency (%)
Age in years (mean ± SD)	26.51 ± 5.15
≤ 20	60 (14.46)
21–30	278 (66.99)
> 30	77 (18.55)
Nationality	
Uganda	397 (95.66)
Kenya	1 (0.24)
Rwanda	14 (3.37)
Congo	3 (0.72)
Education level	
Primary	7 (1.68)
Secondary	104 (25.06)
Tertiary/university	164 (39.52)
No formal education	140 (33.73)
Residence	
Rural	258 (62.17)
Urban	157 (37.83)
Occupation	
House wife	283 (68.17)
Healthcare professional	24 (5.78)
Others	108 (26.02)
Current trimester	
First trimester	134 (32.29)
Second trimester	203 (48.91)
Third trimester	78 (18.79)
Gravida (mean ± SD)	1.84 ± 0.66
1	129 (31.08)
2	224 (53.97)
≥ 3	62 (14.94)
Regular to ANC visits	
Yes	103 (24.82)
No	312 (75.18)
Pregnancy at risk	
Yes	58 (13.97)
No	357 (86.02)
Any stillbirth or miscarriage	
Yes	45 (10.84)
No	370 (89.16)
At least one health problem in the last year	
Yes	26 (6.26)
No	389 (93.73)
At least one medical consultation in the last year	
Yes	23 (5.54)
No	392 (94.45)
Use of medications in previous pregnancy	
Yes	286 (68.91)
No	129 (31.08)
Medical illness in current pregnancy	
Yes	118 (28.43)
No	297 (71.56)
At least one GP visit in the current pregnancy	
Yes	64 (15.42)
No	351 (84.58)
Number of medications used (mean ± SD)	1.85 ± 1.32
0	99 (23.85)
1	47 (11.32)
2	133 (32.01)
≥ 3	136 (32.77)
Under OTC medicine use	
Yes	118 (28.43)
No	297 (71.57)

*Note:* Others = women employed in any private or government organization.

Abbreviations: ANC, antenatal care; GP, general practitioner; OTC, over the counter; SD, standard deviation.

The majority of the women are suffering from anemia (61; 14.69%), malaria (28; 6.75%), and urinary tract infections (18; 1.34%). In our study, the most used medicines during pregnancy are iron and folic acid supplements (312; 75.12%), antibiotics (112; 26.99%), and paracetamol (109; 26.26%). The distribution of medical illnesses and medicines consumed during pregnancy is shown in Table 2.

**Table 2:** Distribution of medical illnesses and medications used during pregnancy (*n* = 415).

Variable	Frequency (%)
Medical illnesses	
Allergy	3 (0.72)
Arthritis	2 (0.49)
Anemia	61 (14.69)
Asthma	5 (1.20)
Cataract	1 (0.24)
Cough	2 (0.48)
Dermatomycosis	2 (0.48)
Diabetes	8 (1.93)
Diarrhea	5 (1.20)
Emesis	4 (0.96)
Epistaxis	1 (0.24)
Gastritis	4 (0.96)
Headache	9 (2.17)
Hypertension	6 (1.44)
Insomnia	2 (0.48)
Joint pain	4 (0.96)
Limb paralysis	1 (0.24)
Malaria	28 (6.75)
Multiple sclerosis	7 (1.69)
Pharyngitis	2 (0.48)
Pneumonia	3 (0.72)
Peptic ulcer disease	5 (1.20)
Sinusitis	1 (0.24)
Snake bite (nonvenomous)	1 (0.24)
Tuberculosis	3 (0.72)
Typhoid	2 (0.48)
Urinary tract infections	18 (1.34)
None	297 (71.57)
Medications used	
Antihistamines	3 (0.72)
Antihypertensives	6 (1.44)
Antidiabetics	8 (1.93)
Paracetamol	109 (26.26)
Antibiotics	112 (26.99)
Antimalarials (prophylactic and therapeutic)	48 (11.56)
Bronchodilators	6 (1.44)
Steroidal hormones	92 (22.17)
NSAIDs	8 (1.93)
Cough suppressants	2 (0.48)
Antitubercular agents	3 (0.72)
Antacids	43 (10.36)
Antiemetics	14 (3.37)
Iron and folic acid	312 (75.12)
None	99 (23.85)

Abbreviation: NSAIDs, nonsteroidal anti‐inflammatory drugs.

More than half of pregnant women are aware of the critical period in which drugs affect the fetus (288; 69.40%), the necessity of modifying the medication regimen during pregnancy (281; 67.71%), and the specific use of each medicine (234; 56.40%). Very few participants accurately identified the name of the medicine they were consuming (117; 28.20%) and medication safety during pregnancy (52; 12.53%). According to the study's findings, most pregnant women interact with their doctor and pharmacist frequently (381; 91.81%), asking about the purpose and safety of the prescription drug (doctor = 287; 69.16%, pharmacist = 351; 84.58%). On the other hand, very few women (123; 29.64%) check the leaflet before consuming medicines. Table 3 presents the distribution of knowledge and medication use practice among pregnant women.

**Table 3:** Knowledge and practices toward safe medication use during pregnancy (*n* = 415).

S. No	Knowledge question	Correct no. (%)
K1	Do you know the name of the medicine(s) that you are currently taking?	117 (28.20)
K2	Do you know the use(s) of all the medicine(s) that you are currently taking?	234 (56.40)
K3	Do you know that some medicines may not be safe during pregnancy?	52 (12.53)
K4	Do you know that some medicines are important to take during pregnancy?	195 (47.00)
K5	Do you know the critical period during pregnancy when medicines are likely to have impact on fetus?	288 (69.40)
K6	Consuming unwanted drugs during pregnancy might have effects for both the mother and the child's health?	193 (46.50)
K7	Medication therapy should be modified during pregnancy if you have a chronic illness.	281 (67.71)
K8	Some medications should never be used in pregnancy regardless of the condition	195 (46.99)
	Practice question	
P1	Do you take medications only after consultation with a doctor or pharmacist?	328 (79.04)
P2	Do you normally check the accompanied leaflet content of the medication?	123 (29.64)
P3	Do you meet your doctor regularly during pregnancy?	381 (91.81)
P4	During prescribing, do you ask the purpose of prescribed medication(s) safety during pregnancy?	287 (69.16)
P5	During dispensing, do you ask the pharmacist how to take dispensed medication(s) and its safety during pregnancy?	351 (84.58)
P6	What is your source of information about medicines?	376 (90.60)

Most (308; 74.22%) of the participants agreed that doctor or pharmacist advice is required to resolve concerns about the use of medications during pregnancy. More than half of the participants hold the belief that stopping medicines can improve the fetus's health (245; 59.04%), that medicine use escalates during pregnancy (278; 66.99%), and that untreated illness can negatively impact fetal development (266; 64.10%). Very few (111; 26.75%) people agree to quit using over‐the‐counter medications during pregnancy. Less than half of the participants thought natural medicines (193; 46.51%), vitamins and supplements (193; 46.51%), and topical treatments (191; 46.02%) were safe during pregnancy. Table 4 shows the distribution of pregnant women's attitude toward medication use.

**Table 4:** Attitude toward safe medication use during pregnancy (*n* = 415).

S. No	Statement	Agree *n* (%)	Uncertain *n* (%)	Disagree *n* (%)
A1	All medicines are harmful to the fetus and pregnant women.	73 (17.59)	108 (26.02)	234 (56.38)
A2	It is better for the fetus if women stop taking medicines during pregnancy.	245 (59.04)	108 (26.02)	62 (14.69)
A3	Pregnant women have a higher threshold for using medicines than normal.	278 (66.99)	46 (11.08)	91 (21.93)
A4	Untreated illness during pregnancy may affect the fetal development.	266 (64.10)	38 (9.16)	111 (26.75)
A5	Natural medicines are considered as safe to use during pregnancy.	193 (46.51)	143 (34.46)	79 (19.04)
A6	Pregnant women should notify their doctor or pharmacist about their concerns and medication use during pregnancy.	308 (74.22)	48 (11.57)	59 (14.22)
A7	Prescription medications are safe to take throughout pregnancy.	195 (46.99)	145 (34.46)	75 (18.07)
A8	Women should stop taking unnecessary OTC medicines during pregnancy.	111 (26.75)	159 (38.31)	145 (34.94)
A9	Vitamins and supplements are considered safe during pregnancy.	193 (46.51)	143 (34.46)	79 (19.04)
A10	Topical medications/preparations are considered safe during pregnancy.	191 (46.02)	141 (33.97)	83 (20.00)

Abbreviation: OTC, over the counter.

The findings of the current study revealed that more than half of the pregnant women have inadequate knowledge (238; 57.4%) and irrational practice (272; 65.5%) toward medication use. Whereas, the majority (326; 78.6%) of the participants have a positive attitude toward safe medication use during pregnancy.

Factors such as urban residence (AOR = 1.54; 95% CI = 2.68–4.49), healthcare profession (AOR = 1.94; 95% CI = 2.36–10.59), regular attendance to ANC visits (AOR = 1.22; 95% CI = 1.06–3.48), visited general practitioner in the current pregnancy (AOR = 2.54; 95% CI = 1.09–5.91), and using at least one medication [one (AOR = 9.00; 95% CI = 2.78–6.43), two (AOR = 5.86; 95% CI = 2.15–15.98), three or more (AOR = 9.88; 95% CI = 3.50–27.87)] were significantly positively associated with adequate knowledge toward safe medication use during pregnancy. Whereas, equal or lower than the secondary education level of the participants was significantly negatively associated with adequate knowledge about safe medication use during pregnancy. In the attitude domain, no factor was significantly associated with the positive attitude toward safe medication use during pregnancy. The practice domain revealed that healthcare profession (AOR = 2.56; 95% CI = 1.86–7.59) regular attendance to ANC visits (AOR = 1.79; 95% CI = 1.05–3.74), and at least one GP visit in the current pregnancy (AOR = 1.73; 95% CI = 1.02–3.25) were positively significantly associated with rational practice toward safe medication use during pregnancy. Whereas age less than 30 years [21–30 years (AOR = 0.53; 95% CI = 0.28–0.98), ≤ 20 years (AOR = 0.12; 95% CI = 0.09–0.26)], and equal or less than secondary educational level [secondary (AOR = 0.23 (95% CI = 0.11–0.50), primary (AOR = 0.12; 95% CI = 0.05–0.33), and no formal education (AOR = 0.12; 95% CI = 0.01–0.26))] were negatively significantly associated with rational practice of safe medication use during pregnancy. The distribution of variables associated with adequate knowledge, positive attitude, and rational practice toward safe medication use during pregnancy is represented in Table 5. A binary and logistic regression analysis of variables predicting adequate knowledge, positive attitude, and rational practice toward safe medication use during pregnancy are presented in Supporting Information S1: Tables S1–S3.

**Table 5:** Binary and multiple logistic regression analysis of variables that predict KAP toward medication use during pregnancy (*n* = 415).

Variable	Adequate knowledge		Positive attitude		Rational practice	
	COR (95% CI), *p* value	AOR (95% CI), *p* value	COR (95% CI), *p* value	AOR (95% CI), *p* value	COR (95% CI), *p* value	AOR (95% CI), *p* value
Age in years						
≤ 20	0.23 (0.10–0.51) ***	0.55 (0.12–2.55)	1.11 (0.45–2.71)		0.50 (0.24–1.03) *	0.12 (0.09–0.26) *
21–30	0.88 (0.53–1.45)	1.05 (0.42–2.64)	0.73 (0.38–1.38)		0.63 (0.37–1.05)	0.53 (0.28–0.98) *
> 30	1.00	1.00	1.00		1.00	1.00
Education level						
No formal education	0.00 (0.00–0.00)	0.00 (0.00–0.00)	1.00 (0.18–5.37)	1.06 (0.17–6.59)	0.09 (0.01–0.84) *	0.12 (0.01–0.26)
Primary	0.02 (0.01–0.04) ***	0.03 (0.01–0.13) ***	2.05 (1.08–3.87) *	2.18 (0.84–5.64)	0.09 (0.05–0.18) ***	0.12 (0.05–0.33)
Secondary	0.08 (0.05–0.15) ***	0.15 (0.01–0.38) ***	1.79 (1.04–3.06) *	1.90 (0.85–4.26)	0.19 (0.11–0.31) ***	0.23 (0.11–0.50)
Tertiary/university	1.00	1.00	1.00	1.00	1.00	1.00
Residence						
Rural	1.00	1.00	1.00	1.00	1.00	1.00
Urban	6.67 (4.29–10.37) ***	1.54 (2.68–4.49) *	0.65 (0.40–1.04)	1.12 (0.58–2.19)	3.53 (2.31–5.40) ***	0.92 (0.48–1.76)
Occupation						
House wife	0.11 (0.07–0.18) ***	0.73 (0.27–1.92)	1.48 (0.88–2.50)	0.82 (0.36–1.85)	0.23 (0.15–0.37)	0.79 (0.37–1.71)
Healthcare professional	3.49 (0.77–15.84)	1.94 (2.36–10.59) *	0.85 (0.32–2.26)	0.93 (0.35–2.49)	3.04 (1.06–8.74) *	2.56 (1.86–7.59) *
Others	1.00	1.00	1.00	1.00	1.00	1.00
Current trimester						
First trimester	1.00	1.00	1.00		1.00	
Second trimester	1.14 (0.73–1.77)	1.350E9 (0.00–€)	1.07 (0.63–1.82)		0.93 (0.59–1.48)	
Third trimester	1.66 (0.94–2.92)	0.29 (0.00–€)	1.12 (0.56–2.21)		1.04 (0.58–1.86)	
Gravida						
1	1.00	1.00	1.00		1.00	
2	1.57 (1.01–2.44) *	a (0.00–€)	0.90 (0.53–1.54)		0.99 (0.63–1.57)	
≥ 3	1.10 (0.59–2.06)	4.89 (0.00–€)	0.86 (0.41–1.80)		0.89 (0.47–1.69)	
Regular to ANC visits						
Yes	7.19 (4.29–12.04) ***	1.22 (1.06–3.48) *	1.01 (0.58–1.73)		2.48 (1.57–3.91) ***	1.79 (1.05–3.74) *
No	1.00	1.00	1.00		1.00	1.00
Pregnancy at risk						
Yes	2.12 (1.21–3.73) **	0.00 (0.00–€)	1.19 (0.59–2.41)		1.53 (0.87–2.69)	0.72 (0.29–1.79)
No	1.00	1.00	1.00		1.00	1.00
Any stillbirth or miscarriage						
Yes	1.20 (0.64–2.23)		1.29 (0.58–2.89)		0.94 (0.49–1.82)	
No	1.00		1.00		1.00	
At least one health problem in the last year						
Yes	1.62 (0.73–3.59)		0.59 (0.25–1.41)		1.20 (0.53–2.72)	
No	1.00		1.00		1.00	
At least one medical consultation in the last year						
Yes	2.18 (0.92–5.17)	1.36 (0.28–6.71)	0.60 (0.24–1.52)		1.24 (0.52–2.93)	
No	1.00	1.00	1.00		1.00	
Use of medications in previous pregnancy						
Yes	1.45 (0.95–2.23)	1.43 (0.34–7.46)	0.89 (0.53–1.49)		0.97 (0.63–1.51)	
No	1.00	1.00	1.00		1.00	
Medical illness						
Yes	0.89 (0.58–1.38)		0.73 (0.44–1.20)		1.02 (0.65–1.59)	
No	1.00		1.00		1.00	
At least one GP visit in the current pregnancy						
Yes	4.29 (2.39–7.71) ***	2.54 (1.09–5.91) *	0.97 (0.51–1.85)		2.33 (1.36–4.00) **	1.73 (1.02–3.25) *
No	1.00	1.00	1.00		1.00	1.00
Number of medications used						
0	1.00	1.00	1.00		1.00	1.00
1	8.19 (3.65–18.40) ***	9.00 (2.68–30.30) ***	1.05 (0.46–2.37)		2.84 (1.37–5.89) **	1.51 (0.65–3.47)
2	4.84 (2.50–9.37) ***	5.86 (2.15–15.98) **	1.71 (0.89–3.28)		1.32 (0.74–2.36)	0.94 (0.48–1.83)
≥ 3	7.92 (4.09–15.32) ***	9.88 (3.50–27.87) ***	0.99 (0.55–1.83)		1.95 (1.10–3.44) *	1.20 (0.63–2.30)

*Note:* Others: women employed in any private or government organization; a = 744821564.081, b = 2429578102.222598, € = 0.9999999.

Abbreviations: ANC, antenatal care; AOR, adjusted odds ratio; COR, crude odds ratio; GP, general practitioner.

*p < 0.05; **p < 0.01; ***p < 0.001.

## 4 Discussion

Global reports have been published on pregnant women's knowledge, attitude, and practice regarding safe medication usage. Understanding pregnant women's KAP regarding medication use and its factors is necessary to provide effective interventions. To the best of our knowledge, this is the first study conducted in Uganda.

The study found that 76.14% of pregnant women take at least one medication, either prescription or non‐prescription. This finding is comparable with the findings of the studies conducted in India (79.62%) and the United Kingdom (76.4%), but lower than the studies conducted in Scotland (85.2%), the United States (82.5%), Malaysia (81.4%), France (89.9%), and Europe, North, and South America, and Australia (81.2%) [3, 17, 35, 36, 37, 38, 39]. Moreover, the prevalence is higher than the findings of studies conducted in Iceland (49.0%), Ireland (46.8%), Canada (27.0%), Ethiopia (64.0%), and Italy (59.6%) [4, 14, 40, 41, 42]. The prevalence of medicine use among pregnant women varies according to studies, depending on medical illnesses, patient type, medication use laws, study design, survey tool used and time of research commencement. In the current study, 28.43% of women are consuming medicines without a prescription. The current study found a low estimate of self‐medication practice when compared to studies conducted in other African nations such as Tanzania (46.24%), Ethiopia (49.2%), Nigeria (31.0%), and Uganda (65.7%) [43, 44, 45, 46]. The majority of pregnant women (71.5%) in the current study does not experience any type of acute or chronic illness during pregnancy, which contributes to the less prevalence of self‐medication practice. Additionally, in the current study 91.81% of the women regularly meet their physician to make decisions regarding medication intake. This approach can promote rational drug use and avoids inappropriate self‐medication practice in pregnancy.

The current study found that the most used drugs among pregnant women are iron and folic acid supplements (75.15%), antibiotics (26.99%), and paracetamol (26.26%). The World Health Organization (WHO) recommends daily iron‐folic acid supplements during pregnancy to avoid maternal anemia and protect neonates from preterm birth, low birth weight, and puerperal sepsis [47]. In our study, excessive usage of iron‐folic acid supplements during pregnancy reveals that healthcare practitioners strictly adhere to nutritional recommendations to avoid issues related to iron‐folic acid deficiency. Antibiotics and paracetamol were found to be the second and third most used medications during pregnancy, respectively. In our analysis, infection exposure during pregnancy was the most common reason for the widespread use of antibiotics and paracetamol. Like our findings, research conducted in Ethiopia (42.50%) and Italy (12.80%) found that antibiotic use is high among pregnant women [14, 48]. According to a study conducted in Saudi Arabia, paracetamol is the most recommended medicine during pregnancy [18]. Changes in co‐morbidities, pregnancy illnesses, antenatal care offered, and drug use policies established in each hospital setting account for the disparity in medication utilization between studies. About 22.17% of pregnant women receive progesterone therapy. This finding contrasted with research conducted in the United States (0.4%–4.5%) and Italy (14.60%) [5, 41]. There was conflicting information regarding the effect of progesterone on improving pregnancy outcomes. As a result, all pregnant women must inquire about evidence‐based information from healthcare experts to avoid misusing medications during pregnancy.

In our study, more than half (57.40%) of the participants showed an inadequate level of knowledge toward safe medication use during pregnancy; similar findings were reported by a study in Malaysia (52.80%) [3]. More than half of the participants are aware of the use of medication they are consuming, the pregnancy period when drugs may affect the fetus, and the modification of the medication regimen required during pregnancy. Many of them were unaware of the medication safety and name of the drug that they were consuming, and taking unwanted medications may cause effects on both mother and fetus. According to a Saudi Arabian study, most pregnant women had a thorough understanding of the recommended and contraindicated medications [18]. Therefore, healthcare professionals and pregnant women need to receive educational interventions focused on medications that are appropriate and contraindicated during pregnancy.

The findings of the binary logistic regression analysis revealed that women living in urban areas, doing healthcare jobs, having regular attendance at the ANC, visiting a GP at least once, and consuming at least one medication were significantly positively associated with adequate knowledge regarding safe medication use during pregnancy. Whereas, women with low educational levels were negatively associated with adequate knowledge of safe medication use during pregnancy. Similar to our study findings, studies conducted in India, Nepal, and Malaysia found that education level and occupation are the most common variables associated with knowledge of medication use during pregnancy [3, 17, 49]. In contrast to our findings, the Indian study also found that variables such as gravida and age were also significantly associated with pregnant women's medication use knowledge levels [17]. The current study findings suggest that women who don't have a formal education and not in the healthcare profession are unable to capture the physician's instructions on safe medication use. Also, women can acquire optimal knowledge about safe medication use if they have regular ANC visits and get advice from the GP if they are suffering from any form of acute or chronic illness during pregnancy. It is a fact that if a woman is under medication therapy, she actively seeks safety information about the medication she is consuming. So, the current study also supports the finding that women who are on medication therapy have adequate knowledge about safe medication use. The low knowledge level of rural women can be justified in the sense that they do not have good access to healthcare facilities or pharmacies to get advice about safe medication use [50].

The current study revealed that most pregnant women had a fair good attitude toward safe medication use. These findings were like the results of studies conducted in India and Nepal [17, 49]. In our study, more than half of the women agreed that doctor advice is mandatory. Though most of the women believed treatment was necessary for medical conditions during pregnancy, they all agreed that stopping medicines can improve fetal health. Evidence shows that women are more restrictive to take medicines during pregnancy [18, 35]. It is necessary to provide appropriate patient counseling especially when medicines are recommended to treat existing or gestational medical illnesses during pregnancy. More than one‐third of the participants perceived those natural medicines, vitamins, supplements, and topical preparation are safe during pregnancy. Evidence was scarce about the safe use of natural medicines during pregnancy [51]. Additionally, most of the literature suggest that natural medicine use can have adverse pregnancy outcomes and there is a need of human studies to support the safe use of natural medicine during pregnancy [52, 53, 54]. So, findings of our study suggest that there is a need for educational interventions targeting the maternal complications associated with the use of natural medicine, supplements, and topical preparations and the importance of the treatment of existing medical illnesses during pregnancy [54].

Though the study shows a high level of positive attitude (78.6%), this was not well correlated with adequate knowledge (42.6%), and rational practice (34.6%) toward safe medication use during pregnancy. The low knowledge and practices about safe medication use over high positive attitude is due to variation in the response scales used for each domain of the KAP. The current study findings inform us that, along with a positive attitude, a high level of good knowledge is required to promote rational practice about safe medication use during pregnancy. In the practice domain, healthcare profession jobs, regular attendance to ANC visits, and at least one GP visit in the current pregnancy were significantly positively associated with rational practice. Whereas, age less than 30 years, and education level less than or equal to secondary level were negatively significantly associated with rational practices. Age, occupation, and educational level were also reported as a contributing factor for rational practice during pregnancy among studies conducted in Nepal and India [17, 49]. These findings suggest that there is a need to enhance women's knowledge levels that can transform their positive attitude into real practice. Evidence shows that the inception of pre‐conception counseling to women can enhance their awareness regarding safe medication during pregnancy [28]. Our study findings suggest that providing appropriate antenatal educational interventions about safe medication use during pregnancy can enhance the knowledge and practices of pregnant women and reduce the drug‐induced maternal and fetal complications.

## 5 Strengths and Limitations

While interpreting the study findings, researchers need to shed light on the following limitations in the context of drug use policy recommendations in pregnancy. Firstly, though the study was an interview‐based survey, response, and reporting bias were inherited in the design of the study due to the subjective nature of participant's opinions. Generally, surveys are limited by response bias, in which participants tend to respond by reporting how they should practice instead of how they practice which can affect the validity of the research. This can Secondly, the cross‐sectional nature of the study design can limit the ability to identify the chronology of events, although several associations were observed. Thirdly, the study was limited to a single hospital that is in the Kyegegwa district in Western Uganda, the generalizability of the findings in other regions of the country may need to be established. The study's findings are not generalizable due to differences in drug usage practices across the country, variations in drug policies in other hospitals, and the types of pregnancy care offered by the hospitals. Fourthly, the questionnaire‐based surveys may restrict participant's answers to the framed questions. Finally, the adoption of a nonrandom convenience sampling technique can result in sampling bias, affecting the generalizability of the study findings. Despite these limitations, there are several strengths to our study, the greatest one is that this is the first Ugandan data regarding knowledge, attitude, and practice of safe medication use during pregnancy. Moreover, the greater response rate and inclusion of the representative sample of the population in our study provide insights into the educational interventions that are required to enhance the KAP regarding safe medication use during pregnancy.

## 6 Conclusion

The study concludes that pregnant women have a positive attitude toward safe medication use; still, there is a wide gap in transforming this positive attitude into rational practice due to a lack of adequate knowledge. Factors like age, education, residence, occupation, antenatal care visit, GP visit, and taking at least one medication were significantly associated with knowledge and or practice regarding safe medication use during pregnancy. So, to address the gap identified in the knowledge and practice of pregnant women, hospital‐based educational interventions (patient counseling, providing medication information leaflet, communicating safety profile) can be initiated targeting young age, lower or no education, rural residents, and participants working in non‐healthcare job. The study suggests that hospital‐based educational interventions should stress the possible negative effects of self‐medication, how important it is to take medications as prescribed, drugs that are contraindicated during pregnancy, screening for potential adverse effects, and the outcomes of drug therapy that can enhance rational medicine use during pregnancy. Also, it is recommended to encourage women to regularly attend their scheduled ANC visits. In case of any medical illness during pregnancy, it is better to receive a general practitioner's advice. For drug safety information, majority of the pregnant women rely on the physician's or pharmacist's advice rather than informal sources like the Internet. So, the physician and pharmacist need combinedly work to improve the KAP of pregnant women on safe medication use.

## Author Contributions

**Musiime Brian:** conceptualization, investigation, methodology, formal analysis, project administration, data curation, resources, writing – original draft, writing – review and editing. **Narayana Goruntla:** conceptualization, investigation, writing – original draft, methodology, writing – review and editing, software, formal analysis, project administration, data curation, supervision, resources. **Bhavana Reddy Bommireddy:** conceptualization, writing – original draft, writing – review and editing, methodology, formal analysis, project administration, investigation, software, data curation, supervision, resources. **Bhavani Mopuri:** conceptualization, investigation, writing – original draft, methodology, writing – review and editing, software, formal analysis, project administration, data curation, supervision, resources. **Easwaran Vigneshwaran:** conceptualization, investigation, writing – original draft, methodology, writing – review and editing, software, formal analysis, project administration, data curation, supervision, resources. **Mohammad Jaffar Sadiq Mantargi:** conceptualization, investigation, writing – original draft, methodology, writing – review and editing, software, formal analysis, project administration, data curation, supervision, resources. **Vishnuvandana Bandaru:** conceptualization, investigation, writing – original draft, methodology, writing – review and editing, software, formal analysis, project administration, data curation, supervision, resources. **Joseph Obiezu Chukwujekwu Ezeonwumelu:** conceptualization, investigation, writing – original draft, methodology, writing – review and editing, software, formal analysis, project administration, data curation, supervision, resources. **Sarad Pawar Naik Bukke:** conceptualization, investigation, writing – original draft, methodology, writing – review and editing, software, formal analysis, project administration, data curation, supervision, resources. **Tadele Mekuriya Yadesa:** conceptualization, investigation, writing – original draft, methodology, writing – review and editing, software, formal analysis, project administration, data curation, supervision, resources. **Ebere Emilia Ayogu:** conceptualization, investigation, writing – original draft, methodology, writing – review and editing, software, formal analysis, project administration, data curation, supervision, resources.

## Disclosure

The authors have nothing to report.

## Ethics Statement

The study protocol, survey instrument, and informed consent process were approved by the Kampala International University—School of Pharmacy Research Committee (KIU SPRC 003/24). The researcher explained the study protocol and objectives to the eligible participants and received oral and written consent voluntarily. All participants had the right to withdraw from the study at any point during the research process (consent, initiation, process, and completion). The study followed the guidelines of the Declaration of Helsinki 1975 for research on human volunteers.

## Conflicts of Interest

The authors declare no conflicts of interest.

## Transparency Statement

The lead author Narayana Goruntla affirms that this manuscript is an honest, accurate, and transparent account of the study being reported; that no important aspects of the study have been omitted; and that any discrepancies from the study as planned (and, if relevant, registered) have been explained.

## Supporting information

Supplementary file.

## Data Availability

The data that support the finding of this study are available from the corresponding author upon reasonable request.
