# Supplementary material for: Knowledge, Attitude, and Practice of Medication Use During Pregnancy: A Cross‐Sectional Study in Western Uganda
Source: Health Sci Rep. 2025 Apr 10;8(4):e70644. doi: 10.1002/hsr2.70644 (PMC11985899; doi:10.1002/hsr2.70644)
Supplement: Supplementary file 1 — Supplementary file. [file HSR2-8-e70644-s001.docx]

**Supplement Table 1: Binary and Multiple Logistic Regression analysis of variables predicting Adequate knowledge toward safe medication use during pregnancy (n=415)**

| Variable | Frequency (%) | Adequate knowledge | | | | |
| --- | --- | --- | --- | --- | --- | --- |
|  |  | Frequency (%)  177 | COR (95% CI)  326 | P – value  143 | AOR (95% CI), P-value | P – value |
| **Demographics** |  |  |  |  |  |  |
| **Age in years** |  |  |  |  |  |  |
| ≤ 20 | 60 (14.46) | 11 (18.3) | 0.23 (0.10-0.51) | <0.001 | 0.55 (0.12-2.55) | 0.448 |
| 21-30 | 278 (66.99) | 128 (46.0) | 0.88 (0.53-1.45) | 0.607 | 1.05 (0.42-2.64) | 0.920 |
| > 30 | 77 (18.55) | 38 (49.3) | 1.00 | 1.00 | 1.00 | 1.00 |
| **Education level** |  |  |  |  |  |  |
| Illiterate | 7 (1.68) | 0 (0.0) | 0.00 (0.00-0.0E0) | 0.999 | 0.00 (0.00-0.0E0) | 0.999 |
| Primary | 104 (25.06) | 9 (8.65) | 0.02 (0.01-0.04) | <0.001 | 0.03 (0.01-0.13) | <0.001 |
| Secondary | 164 (39.52) | 51 (31.09) | 0.08 (0.05-0.15) | <0.001 | 0.15 (0.01-0.38) | <0.001 |
| Tertiary / University | 140 (33.73) | 117 (83.57) | 1.00 | 1.00 | 1.00 | 1.00 |
| **Residence** |  |  |  |  |  |  |
| Rural | 258 (62.17) | 67 (25.96) | 1.00 | 1.00 | 1.00 | 1.00 |
| Urban | 157 (37.83) | 110 (70.06) | 6.67 (4.29-10.37) | <0.001 | 1.54 (2.68-4.49) | 0.032 |
| **Occupation** |  |  |  |  |  |  |
| House wife | 283 (68.17) | 73 (25.79) | 0.11 (0.07-0.18) | <0.001 | 0.73 (0.27-1.92) | 0.521 |
| Healthcare professional | 24 (5.78) | 22 (91.67) | 3.49 (0.77-15.84) | 0.106 | 1.94 (2.36-10.59) | 0.042 |
| Others | 108 (26.02) | 82 (75.92) | 1.00 | 1.00 | 1.00 | 1.00 |
| **Obstetrics** |  |  |  |  |  |  |
| **Current trimester** |  |  |  |  |  |  |
| First trimester | 134 (32.29) | 52 (38.80) | 1.00 | 1.00 | 1.00 | 1.00 |
| Second trimester | 203 (48.91) | 85 (41.87) | 1.14 (0.73-1.77) | 0.575 | 1.350E9 (0.00-€) | 0.999 |
| Third trimester | 78 (18.79) | 40 (51.28) | 1.66 (0.94-2.92) | 0.078 | 0.29 (0.00-€) | 1.000 |
| **Gravida (Mean ± SD)** |  |  |  |  |  |  |
| 1 | 129 (31.08) | 47 (36.43) | 1.00 | 1.00 | 1.00 | 1.00 |
| 2 | 224 (53.97) | 106 (47.32) | 1.57 (1.01-2.44) | 0.047 | a (0.00-€) | 0.999 |
| ≥3 | 62 (14.94) | 24 (38.71) | 1.10 (0.59-2.06) | 0.761 | 4.89 (0.00-€) | 1.000 |
| **Regular to ANC visits** |  |  |  |  |  |  |
| Yes | 103 (24.82) | 79 (76.69) | 7.19 (4.29-12.04) | <0.001 | 1.22 (1.06-3.48) | 0.043 |
| No | 312 (75.18) | 98 (31.41) | 1.00 | 1.00 | 1.00 | 1.00 |
| **Pregnancy at risk** |  |  |  |  |  |  |
| Yes | 58 (13.97) | 34 (58.62) | 2.12 (1.21-3.73) | 0.009 | 0.00 (0.00-€) | 0.997 |
| No | 357 (86.02) | 143 (40.06) | 1.00 | 1.00 | 1.00 | 1.00 |
| **Any still birth or miscarriage** |  |  |  |  |  |  |
| Yes | 45 (10.84) | 21 (46.67) | 1.20 (0.64-2.23) | 0.564 |  |  |
| No | 370 (89.16) | 156 (42.16) | 1.00 | 1.00 |  |  |
| **Clinical** |  |  |  |  |  |  |
| **At least one health problem in the last year** |  |  |  |  |  |  |
| Yes | 26 (6.26) | 14 (53.85) | 1.62 (0.73-3.59) | 0.237 |  |  |
| No | 389 (93.73) | 163 (41.90) | 1.00 | 1.00 |  |  |
| **At least one medical consultation in the last year** |  |  |  |  |  |  |
| Yes | 23 (5.54) | 14 (60.87) | 2.18 (0.92-5.17) | 0.075 | 1.36 (0.28-6.71) | 0.704 |
| No | 392 (94.45) | 163 (41.58) | 1.00 | 1.00 | 1.00 | 1.00 |
| **Use of medications in previous pregnancy** |  |  |  |  |  |  |
| Yes | 286 (68.91) | 130 (45.45) | 1.45 (0.95-2.23) | 0.086 | 1.43 (0.34-7.46) | 0.892 |
| No | 129 (31.08) | 47 (36.43) | 1.00 | 1.00 | 1.00 | 1.00 |
| **Medical illness** |  |  |  |  |  |  |
| Yes | 118 (28.43) | 48 (40.68) | 0.89 (0.58-1.38) | 0.609 |  |  |
| No | 297 (71.56) | 129 (43.43) | 1.00 | 1.00 |  |  |
| **At least one GP visit in the current pregnancy** |  |  |  |  |  |  |
| Yes | 64 (15.42) | 46 (71.87) | 4.29 (2.39-7.71) | <0.001 | 2.54 (1.09-5.91) | 0.030 |
| No | 351 (84.58) | 131 (37.32) | 1.00 | 1.00 | 1.00 | 1.00 |
| **Number of medicines using** |  |  |  |  |  |  |
| **0** | 99 (23.85) | 14 (14.14) | 1.00 | 1.00 | 1.00 | 1.00 |
| 1 | 47 (11.32) | 27 (57.45) | 8.19 (3.65-18.40) | <0.001 | 9.00 (2.68-30.30) | <0.001 |
| 2 | 133 (32.01) | 59 (44.36) | 4.84 (2.50-9.37) | <0.001 | 5.86 (2.15-15.98) | 0.001 |
| ≥3 | 136 (32.77) | 77 (56.62) | 7.92 (4.09-15.32) | <0.001 | 9.88 (3.50-27.87) | <0.001 |

a=744821564.081; b=2429578102.222598; €=0.9999999;

**Supplement Table 2: Binary and Multiple Logistic Regression analysis of variables predicting Positive attitude toward safe medication use during pregnancy (n=415)**

| Variable | Frequency (%) | Positive attitude | | | | |
| --- | --- | --- | --- | --- | --- | --- |
|  |  | Frequency (%) | COR (95% CI) | P – value | AOR (95% CI), P-value | P – value |
| **Age in years** |  |  |  |  |  |  |
| ≤ 20 | 60 (14.46) | 50 (83.33) | 1.11 (0.45-2.71) | 0.817 |  |  |
| 21-30 | 278 (66.99) | 213 (76.62) | 0.73 (0.38-1.38) | 0.331 |  |  |
| > 30 | 77 (18.55) | 63 (81.82) | 1.00 | 1.00 |  |  |
| **Education level** |  |  |  |  |  |  |
| Illiterate | 7 (1.68) | 5 (71.43) | 1.00 (0.18-5.37) | 1.000 | 1.06 (0.17-6.59) | 0.952 |
| Primary | 104 (25.06) | 87 (83.6) | 2.05 (1.08-3.87) | 0.027 | 2.18 (0.84-5.64) | 0.109 |
| Secondary | 164 (39.52) | 134 (81.71) | 1.79 (1.04-3.06) | 0.035 | 1.90 (0.85-4.26) | 0.118 |
| Tertiary / University | 140 (33.73) | 100 (71.43) | 1.00 | 1.00 | 1.00 | 1.00 |
| **Residence** |  |  |  |  |  |  |
| Rural | 258 (62.17) | 210 (81.39) | 1.00 | 1.00 | 1.00 | 1.00 |
| Urban | 157 (37.83) | 116 (73.88) | 0.65 (0.40-1.04) | 0.072 | 1.12 (0.58-2.19) | 0.732 |
| **Occupation** |  |  |  |  |  |  |
| House wife | 283 (68.17) | 229 (80.92) | 1.48 (0.88-2.50) | 0.139 | 0.82 (0.36-1.85) | 0.631 |
| Healthcare professional | 24 (5.78) | 17 (70.83) | 0.85 (0.32-2.26) | 0.745 | 0.93 (0.35-2.49) | 0.888 |
| Others | 108 (26.02) | 80 (74.07) | 1.00 | 1.00 | 1.00 | 1.00 |
| **Current trimester** |  |  |  |  |  |  |
| First trimester | 134 (32.29) | 104 (77.61) | 1.00 | 1.00 |  |  |
| Second trimester | 203 (48.91) | 160 (78.82) | 1.07 (0.63-1.82) | 0.793 |  |  |
| Third trimester | 78 (18.79) | 62 (79.49) | 1.12 (0.56-2.21) | 0.749 |  |  |
| **Gravida (Mean ± SD)** |  |  |  |  |  |  |
| 1 | 129 (31.08) | 103 (79.84) | 1.00 | 1.00 |  |  |
| 2 | 224 (53.97) | 175 (78.12) | 0.90 (0.53-1.54) | 0.704 |  |  |
| ≥3 | 62 (14.94) | 48 (77.42) | 0.86 (0.41-1.80) | 0.700 |  |  |
| **Regular to ANC visits** |  |  |  |  |  |  |
| Yes | 103 (24.82) | 81 (78.64) | 1.01 (0.58-1.73) | 0.980 |  |  |
| No | 312 (75.18) | 245 (78.52) | 1.00 | 1.00 |  |  |
| **Pregnancy at risk** |  |  |  |  |  |  |
| Yes | 58 (13.97) | 47 (81.03) | 1.19 (0.59-2.41) | 0.620 |  |  |
| No | 357 (86.02) | 279 (78.15) | 1.00 | 1.00 |  |  |
| **Any still birth or miscarriage** |  |  |  |  |  |  |
| Yes | 45 (10.84) | 37 (82.22) | 1.29 (0.58-2.89) | 0.526 |  |  |
| No | 370 (89.16) | 289 (78.11) | 1.00 | 1.00 |  |  |
| **At least one health problem in the last year** |  |  |  |  |  |  |
| Yes | 26 (6.26) | 18 (69.23) | 0.59 (0.25-1.41) | 0.236 |  |  |
| No | 389 (93.73) | 308 (79.18) | 1.00 | 1.00 |  |  |
| **At least one medical consultation in the last year** |  |  |  |  |  |  |
| Yes | 23 (5.54) | 16 (69.56) | 0.60 (0.24-1.52) | 0.284 |  |  |
| No | 392 (94.45) | 310 (79.08) | 1.00 | 1.00 |  |  |
| **Use of medications in previous pregnancy** |  |  |  |  |  |  |
| Yes | 286 (68.91) | 223 (77.97) | 0.89 (0.53-1.49) | 0.667 |  |  |
| No | 129 (31.08) | 103 (79.84) | 1.00 | 1.00 |  |  |
| **Medical illness** |  |  |  |  |  |  |
| Yes | 118 (28.43) | 88 (74.58) | 0.73 (0.44-1.20) | 0.214 |  |  |
| No | 297 (71.56) | 238 (80.13) | 1.00 | 1.00 |  |  |
| **At least one GP visit in the current pregnancy** |  |  |  |  |  |  |
| Yes | 64 (15.42) | 50 (78.12) | 0.97 (0.51-1.85) | 0.928 |  |  |
| No | 351 (84.58) | 276 (78.63) | 1.00 | 1.00 |  |  |
| **Number of medicines using** |  |  |  |  |  |  |
| **0** | 99 (23.85) | 75 (75.76) | 1.00 | 1.00 |  |  |
| 1 | 47 (11.32) | 36 (76.59) | 1.05 (0.46-2.37) | 0.912 |  |  |
| 2 | 133 (32.01) | 112 (84.21) | 1.71 (0.89-3.28) | 0.109 |  |  |
| ≥3 | 136 (32.77) | 103 (75.73) | 0.99 (0.55-1.83) | 0.997 |  |  |

**Supplement Table 3: Binary and Multiple Logistic Regression analysis of variables predicting Rational practice toward safe medication use during pregnancy (n=415)**

| Variable | Frequency (%) | Rational practice | | | | |
| --- | --- | --- | --- | --- | --- | --- |
|  |  | Frequency (%) | COR (95% CI) | P – value | AOR (95% CI), P-value | P – value |
| **Age in years** |  |  |  |  |  |  |
| ≤ 20 | 60 (14.46) | 17 (28.3) | 0.50 (0.24-1.03) | 0.049 | 0.12 (0.09-0.26) | 0.034 |
| 21-30 | 278 (66.99) | 92 (33.09) | 0.63 (0.37-1.05) | 0.074 | 0.53 (0.28-0.98) | 0.042 |
| > 30 | 77 (18.55) | 34 (44.15) | 1.00 | 1.00 | 1.00 | 1.00 |
| **Education level** |  |  |  |  |  |  |
| Illiterate | 7 (1.68) | 1 (14.28) | 0.09 (0.01-0.84) | 0.034 | 0.12 (0.01-0.26) | 0.048 |
| Primary | 104 (25.06) | 14 (13.46) | 0.09 (0.05-0.18) | <0.001 | 0.12 (0.05-0.33) | <0.001 |
| Secondary | 164 (39.52) | 40 (24.39) | 0.19 (0.11-0.31) | <0.001 | 0.23 (0.11-0.50) | <0.001 |
| Tertiary / University | 140 (33.73) | 88 (62.85) | 1.00 | 1.00 | 1.00 | 1.00 |
| **Residence** |  |  |  |  |  |  |
| Rural | 258 (62.17) | 61 (23.64) | 1.00 | 1.00 | 1.00 | 1.00 |
| Urban | 157 (37.83) | 82 (52.22) | 3.53 (2.31-5.40) | <0.001 | 0.92 (0.48-1.76) | 0.801 |
| **Occupation** |  |  |  |  |  |  |
| House wife | 283 (68.17) | 64 (22.61) | 0.23 (0.15-0.37) | <0.001 | 0.79 (0.37-1.71) | 0.550 |
| Healthcare professional | 24 (5.78) | 19 (79.17) | 3.04 (1.06-8.74) | 0.039 | 2.56 (1.86-7.59) | 0.041 |
| Others | 108 (26.02) | 60 (55.55) | 1.00 | 1.00 | 1.00 | 1.00 |
| **Current trimester** |  |  |  |  |  |  |
| First trimester | 134 (32.29) | 47 (35.07) | 1.00 | 1.00 |  |  |
| Second trimester | 203 (48.91) | 68 (33.50) | 0.93 (0.59-1.48) | 0.765 |  |  |
| Third trimester | 78 (18.79) | 28 (35.90) | 1.04 (0.58-1.86) | 0.904 |  |  |
| **Gravida (Mean ± SD)** |  |  |  |  |  |  |
| 1 | 129 (31.08) | 45 (34.88) | 1.00 | 1.00 |  |  |
| 2 | 224 (53.97) | 78 (34.82) | 0.99 (0.63-1.57) | 0.991 |  |  |
| ≥3 | 62 (14.94) | 20 (32.26) | 0.89 (0.47-1.69) | 0.720 |  |  |
| **Regular to ANC visits** |  |  |  |  |  |  |
| Yes | 103 (24.82) | 52 (50.48) | 2.48 (1.57-3.91) | <0.001 | 1.79 (1.05-3.74) | 0.032 |
| No | 312 (75.18) | 91 (29.17) | 1.00 | 1.00 | 1.00 | 1.00 |
| **Pregnancy at risk** |  |  |  |  |  |  |
| Yes | 58 (13.97) | 25 (43.10) | 1.53 (0.87-2.69) | 0.137 | 0.72 (0.29-1.79) | 0.476 |
| No | 357 (86.02) | 118 (33.05) | 1.00 | 1.00 | 1.00 | 1.00 |
| **Any still birth or miscarriage** |  |  |  |  |  |  |
| Yes | 45 (10.84) | 15 (33.33) | 0.94 (0.49-1.82) | 0.867 |  |  |
| No | 370 (89.16) | 128 (34.59) | 1.00 | 1.00 |  |  |
| **At least one health problem in the last year** |  |  |  |  |  |  |
| Yes | 26 (6.26) | 10 (38.46) | 1.20 (0.53-2.72) | 0.658 |  |  |
| No | 389 (93.73) | 133 (34.19) | 1.00 | 1.00 |  |  |
| **At least one medical consultation in the last year** |  |  |  |  |  |  |
| Yes | 23 (5.54) | 9 (39.13) | 1.24 (0.52-2.93) | 0.628 |  |  |
| No | 392 (94.45) | 134 (34.18) | 1.00 | 1.00 |  |  |
| **Use of medications in previous pregnancy** |  |  |  |  |  |  |
| Yes | 286 (68.91) | 98 (34.26) | 0.97 (0.63-1.51) | 0.902 |  |  |
| No | 129 (31.08) | 45 (34.88) | 1.00 | 1.00 |  |  |
| **Medical illness** |  |  |  |  |  |  |
| Yes | 118 (28.43) | 41 (34.74) | 1.02 (0.65-1.59) | 0.938 |  |  |
| No | 297 (71.56) | 102 (34.34) | 1.00 | 1.00 |  |  |
| **At least one GP visit in the current pregnancy** |  |  |  |  |  |  |
| Yes | 64 (15.42) | 33 (51.56) | 2.33 (1.36-4.00) | 0.002 | 1.73 (1.02-3.25) | 0.041 |
| No | 351 (84.58) | 110 (31.34) | 1.00 | 1.00 | 1.00 | 1.00 |
| **Number of medicines using** |  |  |  |  |  |  |
| **0** | 99 (23.85) | 25 (25.25) | 1.00 | 1.00 | 1.00 | 1.00 |
| 1 | 47 (11.32) | 23 (48.94) | 2.84 (1.37-5.89) | 0.005 | 1.51 (0.65-3.47) | 0.334 |
| 2 | 133 (32.01) | 41 (30.83) | 1.32 (0.74-2.36) | 0.353 | 0.94 (0.48-1.83) | 0.855 |
| ≥3 | 136 (32.77) | 54 (39.70) | 1.95 (1.10-3.44) | 0.021 | 1.20 (0.63-2.30) | 0.581 |
